# Orthonormal Bernstein Galerkin technique for computations of higher order eigenvalue problems

**DOI:** 10.1016/j.mex.2023.102006

**Published:** 2023-01-05

**Authors:** Humaira Farzana, Samir Kumar Bhowmik, Md. Shafiqul Islam

**Affiliations:** a Department of Arts & Sciences, Ahsanullah University of Sciences & Technology, Dhaka 1215, Bangladesh; b Department of Mathematics, University of Dhaka, Dhaka 1000, Bangladesh; c Department of Applied Mathematics, University of Dhaka, Dhaka 1000, Bangladesh

**Keywords:** Galerkin MWR, Orthonormal Bernstein polynomials, Eigenvalue/Rayleigh numbers, Galerkin Finite Element Method

## Abstract

The numerical approximation of eigenvalues of higher even order boundary value problems has sparked a lot of interest in recent years. However, it is always difficult to deal with higher-order BVPs because of the presence of boundary conditions. The objective of this work is to investigate a few higher order eigenvalue (Rayleigh numbers) problems utilizing the method of Galerkin weighted residual (MWR) and the effect of solution due to direct implementation of polynomial bases. The proposed method develops a precise matrix formulation for the eighth order eigenvalue and linear electro-hydrodynamic (EHD) stability problems.

• The article explores the same for tenth and twelfth order eigenvalue problems.
• This method involves computing numerical eigenvalues using Bernstein polynomials as the basis functions.
• The novel weighted residual Galerkin technique's performance is numerically validated by comparing it to other numerical/analytical approaches in the literature.

## Graphical abstract

**Figure:**
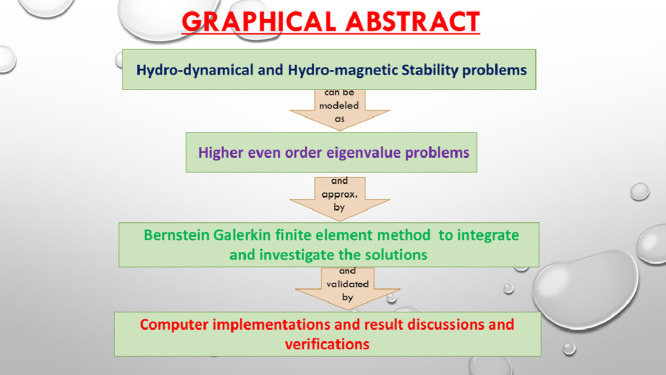

Specifications Table

**Table:** 

Subject area:	Engineering
More specific subject area:	Computational methods for Mathematical Models
Name of your method:	Galerkin Finite Element Method
Name and reference of original method:	*Orthonormal Bernstein Galerkin Technique*
Resource availability:	References listed below

## Introduction

Higher even order BVPs occur in various physical and biological problems. Hydrodynamics, hydro magnetic stability and astrophysics are a few to name. Many researchers have investigated higher even order BVPs due to their scientific significance and applications in a variety of fields of applible branches of sciences. Based on the literature review, many researchers have attempted to address higher order boundary value problems. Nevertheless, only a few studies have investigated higher order differential eigenvalue problems that arise in hydrodynamic and hydro-magnetic stability theory. Eighth, tenth, and twelfth order eigenvalues have endured extensive numerical computation in [1,2]. Additionally, the authors [3] have explored an eigenvalue problem in EHD that indicates the presence of electric forces and is modeled by an 8th order differential operator. The explicit use of numerical methods in some of these scenarios may produce erroneous results because of the bifurcation issues with the stable solutions of well-known Navier-Stokes equations (or of certain existing models). It is to be noted that bifurcations occur as the eigenvalues depend on various physical parameters. Consequently, an analytical solutions as well as numerical investigation of hydrodynamic stability theory is greatly desired.

When the bottom layer of fluid is highly agitated underside under the influence of rotation, the numerical computation of eighth, tenth, and twelfth order [1], [2], [3] eigenvalues occur. The top layered fluid will be heavier than that of the fluid at the bottom and in this state, which causes the layers to be potentially unstable. Thus, viscosity on the part of the fluid has a prevent tendency to rearrange itself. In this work, we are influenced by any supplementary effects of spins, and their rotation will initiate new components into the resulting thermal instability. In fluid flow, instability becomes entrenched typically as over stability in the presence of rotation, but it continues as stationary convection due to the impact of a magnetic field [1,2]. When the instability of a magnetic field manifests as over stability, eighth order differential eigenvalue difficulties arise [1] and can be illustrated in the following form:

(1) $$\left(D^{2}-a^{2}-p_{1}\sigma \right)\left[{\left(D^{2}-a^{2}-\sigma \right)}^{2}\left(D^{2}-a^{2}\right)+TD^{2}\right]u\left(x\right)=-Ra^{2}\left(D^{2}-a^{2}-\sigma \right)u\left(x\right),$$

(1a) u(x)→0asx→∞.

The Rayleigh critical value, which is the smallest eigenvalue, changes as gravity changes [2]. In Straughan's monograph [4], the hydrodynamic stability equations are described in further detail. The lowest possible value of R and aattained by solving Eqs. (1) and (1a) for the matched value of σ, which may be complex, it is supposed that σ, R is complex. However, for the Engineering significance of R, compels it to be real and subsequently σ(=iμ) is purely imaginary [1].

When a homogeneous magnetic field over the fluid in the same direction as gravity induces ordinary convection and over stability, respectively, tenth and twelfth order differential eigenvalue issues [1], [2] arise. The essential condition for stability is popularly known as Rayleigh criterion. Tenth order differential eigenvalue problem which occurs as instability is of the form [1].

(2) $$\left(D^{2}-a^{2}\right)\left[{\left\{{\left(D^{2}-a^{2}\right)}^{2}-QD^{2}\right\}}^{2}+TD^{2}\left(D^{2}-a^{2}\right)\right]u\left(x\right)+Ra^{2}\left[{\left(D^{2}-a^{2}\right)}^{2}-QD^{2}\right]u\left(x\right)=0.$$

Twelfth order equation takes the form [1]

(3) $$\begin{array}{ccc} & & \left(D^{2}-a^{2}-p_{1}\sigma \right)\left[\left(D^{2}-a^{2}\right){\left\{\left(D^{2}-a^{2}-\sigma \right)\left(D^{2}-a^{2}-p_{2}\sigma \right)-QD^{2}\right\}}^{2}+\;TD^{2}{\left(D^{2}-a^{2}-\;\;p_{2}\sigma \right)}^{2}\right]u\left(x\right)\\ & & \;+Ra^{2}\left[\left(D^{2}-a^{2}-\sigma \right)\left(D^{2}-a^{2}-p_{2}\sigma \right)-QD^{2}\right]\left(D^{2}-a^{2}-p_{2}\sigma \right)u\left(x\right)=0. \end{array}$$

The stability of fluid flow is determined by numerical value of the non-dimensional parameters, referred to as Taylor numbers which gives a measure of extent to which Rayleigh's criteria is violated.

This study utilizes a set of orthonormal Bernstein polynomials over a finite domain. It is tempting to implement due to the convergence of Bernstein polynomials for specific features, as well as non-negative optimal stable bases among other bases [5], [6], [7], [8], [9]. Considering functions of bounded variation, the authors of the study [5] estimated the convergence rate of Bernstein polynomials in connection with the arithmetic means through the series of overall variation. Several researchers have used Bernstein polynomials in recent years as a practical tool because of their optimal stable bases among nonnegative bases and their dependability for a variety of noteworthy features used in computer-aided geometric design [5]. The authors [6] employed operational matrices of Bernstein polynomials and its expansion to solve parabolic PDEs. The studies [7,8], provide numerous intuitive geometrical qualities, elegant algorithms, and great numerical stability under the veil of Bézier curve and surface representations [9] gives an overview of Bernstein polynomial applications and illustrates how the Galerkin method is used to solve high even-order differential equations.

In general, an even order Benard type convection problems (the 2*k*-th order, *k*= 4, 5, 6) together with homogeneous BCs can be written as [9]

(4) $$u^{\left(2k\right)}+\sum_{i=1}^{2k-1}\rho_{i}\left(x\right)u^{\left(i\right)}+\lambda \rho_{0}u=0,$$

If the stated boundary conditions below are met

(4a) $$u^{\left(n\right)}\left(0\right)=0,\;u^{\left(n\right)}\left(1\right)=0,\;0\leq n\leq k-1.$$

There are a few works of literature on the approximate eigenvalues of higher order Sturm-Liouville problems. The authors [1] focused on two numerical approaches of finite difference method, namely direct numerical technique, second and fourth-order finite difference techniques to compute eigenvalue problems illustrated therein. In [1], they demonstrated that their direct numerical approach (without reducing the higher order derivatives) eventually brings an ill-conditioned system and undergoes word length difficulties as well. The authors updated the various existing software to compute the eigenvalues with good accuracy. For the specified values of the parameters(T,a,μ), the computed each eigenvalue R becomes complex. The authors varied *a* and μto minimize the imaginary part. Besides, direct methods cause some difficulties due to software implementations. As a result, some infinite eigenvalues occur. However, their second approach avoids this difficulty by giving complex banded coefficient matrices, but much more expensive to implement. From these discussions, we aim to develop a simple and efficient technique which works well for these kinds of problems with less difficulties. The thermal instability of fluid flow arises in sixth order Benard convection problems and Rayleigh numbers have been studied thoroughly in [[10], [11], [12]] applying numerous spectral techniques.

Several numerical methods, namely local adaptive differential quadrature have been implemented in various studies. A numerical method namely Adomian decomposition methods [13,14], energy inequalities method [15], Galerkin based Septic B-Splines technique [16], splines and non-polynomial spline techniques [17], [18], [19], Local adaptive differential quadrature method [20], reproducing kernel Hilbert space method [21] to solve the boundary value problems of higher order with multi-boundary conditions. The authors of [3] used both analytical and numerical methods to investigate the linear Electro-hydrodynamic stability problem of an eighth order differential equation. The latter one is implemented through spectral Galerkin and collocation methods taking Chebyshev and Legendre polynomials [12] as basis functions. The rigorous study of eighth, tenth, and twelfth order linear and nonlinear BVPs using Galerkin MWR and polynomials as the basis function is found in [22], [23], [24], [25]. The numerical approximations on eighth, tenth and twelfth order eigenvalue problem is infrequent. Therefore, we focused on an efficient numerical approach for solving equations analogous to (4). A general approach to solve an even-order Sturm-Liouville problem has recently been developed [26] using the Lie group method and the Magnus expansion (tested for up to eight). Thus to the best of our knowledge a general higher even order such problems has not been studied before using Orthonormal Bernstein Galerkin Technique. This fact motivates us to study further focusing Eq. (4) using Bernstein Galerkin Technique to test the efficiency of considering such polynomials.

In the proposed method, basis functions are satisfied by the Dirichlet-type boundary conditions. On the other hand, in the weak form of the Galerkin residual equation, all the essential types of boundary conditions are incorporated directly. The vanishing at the two boundaries of the interval gives greater flexibility to the Bernstein polynomials and is found to be more attractive for implementing the Galerkin method of weighted residual. Difference schemes are used in [27] to solve the one-dimensional wave equation with nonlocal boundary conditions. The circuit's time and frequency domain properties are examined [28], the filter parameters are chosen analytically, and the outcomes are numerically tested.

The rest of the article is organized as follows:

Several well-known properties of Bernstein polynomials shifted orthonormal Bernstein polynomials are listed in section 2.
Section 3 illustrates the Bernstein Galerkin method to solve higher even order eigenvalue problems. Section 3 further explores error estimation and the convergence of the residual function.
To confirm the reliability and proficiency of the presented method, several numerical examples of BVPs, available in the literature, are presented in Section 4.
Section 5 of this study mentions its findings as a short conclusion.

## Piecewise polynomial basis functions

### Bernstein polynomials

Even though Bernstein polynomials have numerous advantageous characteristics, they lack the attribute of orthogonality. The orthogonality property is particularly helpful for many applications, including least squares approximation and finite element methods. As a result, Bernstein polynomials are frequently less practical to use in these methods than conventional orthogonal polynomials like Legendre, Chebyshev, or Jacobi polynomials.

The optimal stable, nonnegative Bernstein polynomial basis gained popularity among several researchers and useful properties have been applied to solve regular as well as singular second to higher order BVPs and eigenvalue problems [[9], [10], [11],^22^,^23^,[29], [30], [31], [32], [33], [34], [35], [36], [37], [38]]. As stated in [22], the polynomials can be used to form a basis over [0, 1] which is complete. Following are the specifications for Bernstein polynomials over the finite range [0, 1]

(5) $$\;b_{\;i,n}\left(x\right)=\left(\begin{array}{c} n\\ i \end{array}\right)x^{i}{\left(1-x\right)}^{n-i},\;0\leq i\leq n,$$

where *n* being positive integer and $\left(\begin{array}{c} n\\ i \end{array}\right)=\frac{n!}{i!(n-i)!}.$

A few important and useful properties of Bernstein polynomials

(i) $b_{i,n}\left(x\right)=0,\;\text{if}\;i\langle 0\;\;or\;i\rangle n.$
(ii) $\sum_{i=0}^{n}b_{i,n}\left(x\right)=1.$
(iii) All Bernstein polynomials are zero at the end points in [0, 1], that is,

$\;b_{i,n}\left(0\right)=0$ and $b_{i,n}\left(1\right)=0$, i=1,2,3..........,n−1.

(iv) $\left(n+1\right)b_{i,n}\left(x\right)=\left(n-i+1\right)b_{i,n+1}\left(x\right)\;+\left(i+1\right)b_{i+1,n+1}\left(x\right)$
(v) The *n*-th derivative of Bernstein basis can be stated as
  $$b{{}^{\prime }}_{i,n}\left(x\right)=n\left[b_{i-1,n-1}\left(x\right)-b_{i,n-1}\left(x\right)\right]$$
(vi) The binomial expansion of the nth degree Bernstein polynomial can be simply expressed in terms of any higher power base

$\;b_{i,n}\left(x\right)=\sum_{j=0}^{n-i}{\left(-1\right)}^{j}\;\left(\begin{array}{c} n\\ i \end{array}\right)\left(\begin{array}{c} n-i\\ j \end{array}\right)x^{i+j},\;x\in \left[0,1\right]$ where ${\left(1-x\right)}^{n-i}=\sum_{j=0}^{n-i}{\left(-1\right)}^{j}\;\left(\begin{array}{c} n-i\\ j \end{array}\right)x^{j}$,

which follows that $b_{i,n}\left(x\right)=\sum_{j=i}^{n}{\left(-1\right)}^{j-i}\left(\begin{array}{c} n\\ i \end{array}\right)\;\left(\begin{array}{c} n-i\\ j-i \end{array}\right)x^{j},$ [32] for i=0,1,2,3,.......,n.

### Shifted orthonormal Bernstein polynomials

Orthonormal Bernstein polynomials over [0, 1] can be explicitly represented by utilizing the Gram-Schmidt procedure on the set of Bernstein polynomials in Eq. (3) of different degree *n* is given as

(6) $$B_{i,n}\left(x\right)=\sqrt{2\left(n-i\right)+1}\;{\left(1-x\right)}^{n-i}\sum_{k=0}^{n}{\left(-1\right)}^{k}\left(\begin{array}{c} 2n+1-k\\ i-k \end{array}\right)\left(\begin{array}{c} i\\ k \end{array}\right)x^{i-k}$$

where ${\left(1-x\right)}^{n-i}=\sum_{j=0}^{n-i}{\left(-1\right)}^{j}\left(\begin{array}{c} n-i\\ j \end{array}\right)x^{j}$,i=0,1,2,3,.......,n.

Utilizing Bernstein non-orthonormal basis, the above expression takes the form

(7) $$\;B_{i,n}\left(x\right)=\sqrt{2\left(n-i\right)+1}\sum_{k=0}^{n}{\left(-1\right)}^{k}\frac{\left(\begin{array}{c} 2n+1-k\\ k \end{array}\right)\left(\begin{array}{c} i\\ k \end{array}\right)}{\left(\begin{array}{c} n-k\\ i-k \end{array}\right)}b_{i-k,n-k}$$

The orthonormal Bernstein polynomial $B_{i,n}\left(x\right)$ is the n th eigenfunction of the singular Sturm Liouville problems

$$\frac{d}{dx}\left[x{\left(1-x\right)}^{2}\frac{dB\left(x\right)}{dx}\right]+n\left(n+2\right)\left(1-x\right)\frac{dB\left(x\right)}{dx}+\left(n-k+1\right)\left(k-n\right)B\left(x\right)=0.$$

In terms of the weight function, these polynomials have an orthogonality relationship i.e.,θ(x)=1.

(8) $${\left(B_{i,n},B_{j,n}\right)}_{\theta \left(x\right)}=\int_{0}^{1}B_{i,n}\left(x\right)B_{j,n}\;\left(x\right)\theta \left(x\right)dx=\delta_{ij},$$

where

$$\delta_{i,j}=\left\{ \begin{array}{c} 1,\;\;i=j\\ 0,\;\;i\neq j\;. \end{array}\right.$$

The orthonormal Bernstein polynomials necessarily satisfy the following relationships over the interval [0,1]

$$\int_{0}^{1}B_{i,n}\left(x\right)B_{j,n}\left(x\right)dx=\sqrt{2\left(n-i\right)+1}\;\sum_{k=0}^{i}{\left(-1\right)}^{k}\frac{\left(\begin{array}{c} 2n+1-k\\ i-k \end{array}\right)\left(\begin{array}{c} i\\ k \end{array}\right)\left(\begin{array}{c} n\\ j \end{array}\right)}{\left[2n+1-k\right]\left(\begin{array}{c} 2n-k\\ \;i+j-k\; \end{array}\right)};$$

k=0,1,2,…..i,j=0,1,2,…,n&j<i.

## Orthonormal Bernstein-Galerkin technique

Here we move on to the main goal and focus on our key issues in this section. To be specific we are now interested in finding the solution to Eq. (4) that also satisfies boundary condition 4(a) using Bernstein polynomials. The integrals to the higher order eigenvalue problem defined in (4) is considered here.

(9) $$\text{Let}\;V_{n}=\text{span}\left\{B_{0,n}\left(x\right),\;B_{1,n}\left(x\right),\;B_{2,n}\left(x\right)........B_{n,n}\left(x\right)\right\},\;0\leq m\leq q-1,$$

(9a) $$U_{n}=\left\{w\in V_{n}:\;\;w^{\left(m\right)}\left(0\right)=w^{\left(m\right)}\left(1\right)=0\right\},\;0\leq m\leq q-1.$$

The normalized Bernstein Galerkin approximation [9] to the Eq. (4) is to obtain $u_{n}\in U_{n}\;$in a way such that,

(10) $$\left(u_{n}^{\left(2q\right)},w_{n}\right)+\sum_{i=1}^{2q-1}\rho_{i}\left(x\right)\left(u_{n}^{\left(i\right)},w_{n}\right)+\lambda \rho_{0}\left(u_{n},w_{n}\right)=0,\;\forall \;w_{n}\in U_{n},$$

where the inner product$\;\langle u,w\rangle =\int_{I}u\left(x\right)w\left(x\right)\theta \left(x\right)dx$ is defined as in $L^{2}\left(I\right)$ and its norm

$$u{\left(x\right)}^{2}=\int_{I}u^{2}\left(x\right)\theta \left(x\right)dx.$$

It is of utmost importance to highlight that choosing a suitable basis for $U_{n}$ to achieve the Bernstein-Galerkin approximation to (10) that yields the simplest linear system is the key challenge in using the Galerkin Bernstein method of weighted residual. An appropriate normalized basis$\;\varphi_{j}$ for Galerkin MWR can be written as

(11) $$\;\varphi_{j}\left(x\right)=B_{j,n}\left(x\right),$$

where $\varphi_{i}\left(x\right)\;\epsilon \;U_{n}\;$for all i=k,k+1,.....,n−k. Because of the 2k boundary conditions, the first and last k expansion coefficients are both zero.

Consequently, for n≥2q, we have

(12) $$U_{n}=span\left\{\varphi_{q}\left(x\right),\varphi_{q+1}\left(x\right),\varphi_{q+2}\left(x\right)............,\varphi_{n-q}\left(x\right)\right\}$$

which leads to

(13) $$\left(u_{n}^{\left(2q\right)},\varphi_{j}\left(x\right)\right)+\sum_{i=1}^{2q-1}\rho_{i}\left(x\right)\left(u_{n}^{\left(i\right)},\varphi_{j}\left(x\right)\right)+\lambda \rho_{0}\left(u_{n},\varphi_{j}\left(x\right)\right)=0.$$

∀j∈q,q+1,q+2........,n−q.

(14) $$\text{Suppose}\;u_{n}\left(x\right)=\sum_{j=q}^{n-q}c_{j}\varphi_{j}\left(x\right),\;\xi ={\left(c_{q},c_{q+1},c_{q+2},......,c_{n-q}\right)}^{T},$$

$$C=\left(c_{jk}\right),\;\;G_{i}=\left(g_{jk}^{i}\right),\;q\leq j,\;k\leq n-q$$

where

$$\;c_{jk}=\langle B_{k,n}^{\left(2q\right)},B_{j,n}\rangle =\int_{0}^{1}B_{k,n}^{\left(2q\right)}\left(x\right)B_{j,n}\left(x\right)dx,$$

$$\;g_{jk}^{i}=\langle B_{k,n}^{\left(i\right)},B_{j,n}\rangle =\int_{0}^{1}B_{k,n}^{\left(i\right)}\left(x\right)B_{j,n}\left(x\right)dx,$$

$$g_{jk}^{0}=B_{k,n},B_{j,n}=\int_{0}^{1}B_{k,n}\left(x\right)B_{j,n}\left(x\right)dx.$$

Thus Eq. (13) takes the form

(15) $$\left(C+\sum_{i=1}^{2q-1}\rho_{i}G_{i}\;+\lambda \rho_{0}G_{0}\right)\xi =0,\;\xi \neq 0$$

where the matrix components $C,G_{i},$ and $G_{0},\;i=1,2,3,\ldots \ldots ..,\;2q-1\;$are illustrated in detail [9].

Thus Eq. (15) is comparable to the following matrix equation

(16) F+λG=0,

where

(16a) $$F=C+\sum_{i=1}^{2q-1}\rho_{i}G_{i}\;\text{and}\;\rho_{0}G_{0}=G.$$

Finally, the eigenvalues are obtained in solving the system (16) as

(17) $$\lambda I=-FG^{-1}.$$

### Convergence of Galerkin MWR

Stability and convergence measures [9] for the polynomial estimations in the Galerkin MWR have been studied by several authors [9,10,[22], [23], [24], [25],[31], [32], [33]]. Moreover, orthogonal polynomials basis can approximate general functions in Hilbert spaces. Here, we reveal a short appraisal on this topic.

Function approximation:

We define $L^{2}\left[0,1\right]=\left\{f:\;[0,1]\to R|\;f\;is\;\text{measurable}\;\text{and}\;∥f∥<\infty \;\right\}$ .

Here we recall Bessel inequality ${\left(\int_{0}^{1}{\left|f(x)\right|}^{2}dx\right)}^{\frac{1}{2}}$ is the norm induced by the inner product

(18) $$\langle f,\psi \rangle =\int_{0}^{1}f\left(x\right)\psi \left(x\right)dx.$$

**Theorem:** (Bessel inequality) Let$\;(\;\varphi_{k})\;$be an orthonormal sequence in an inner product space i.e., in H, then for every f∈H

$$\sum_{k=1}^{n}{\left|\langle f,\varphi_{k}\;\rangle \right|}^{2}\leq \sum_{k=1}^{n}\left|f,f\right|\leq f^{2}.$$

$H=L^{2}\left[0,1\right]\;\text{and}\;\left\{B_{0,n}\left(x\right),\;B_{1,n}\left(x\right),\;B_{2,n}\left(x\right)........B_{n,n}\left(x\right)\right\}\subset H$ be set of orthonormal Bernstein polynomials of order n where the polynomial basis span in $V_{n}$ as defined by Eq. (9).

We can define as $\;P_{n}:\;L^{2}\left[0,1\right]\to V_{n}$

**by**

$$\;P_{n}f\left(x\right)=\sum_{i=0}^{n}c_{i}B_{i,n}\left(x\right),\;c_{i}\;\mathrm{are}\;\mathrm{constants}\;\mathrm{to}\;\mathrm{be}\;\mathrm{determined}.$$

Theorem [39]: Let f be an arbitrary element in H. Since $V_{n}$ is a finite dimensional complete and closed subspace, therefore, $V_{n}$ is a subset of H. Therefore, f has the best unique approximation out of $V_{n}$, say $g\in V_{n}\;such\;that\;∥f-g{∥}_{2}\leq ∥f-v{∥}_{2}\;\forall \;v\in V_{n}$ where $∥f{∥}_{2}=\sqrt{\langle f,\;f\rangle }$.

Since, $g\in V_{n}$, there exists a unique coefficient $c_{i},\;i=0,1,2,3,.............,n$ such that

$f\approx g=\sum_{i=0}^{n}c_{i}B_{i,n}\left(x\right)=C^{T}\varphi \left(x\right)$, where $\varphi^{T}=\left[B_{0,n},B_{1,n},\;B_{2,n}\ldots \ldots ..,B_{n,n}\right]$ and

$C^{T}=\left[c_{1},c_{2},\ldots \ldots .,c_{n}\right]$ where $C^{T}\langle \varphi ,\varphi \rangle =\;f\left(x\right),\varphi \left(x\right).$

**Lemma:** [6,10]**:** Suppose that the function f:[0,1]→R is n+1 times continuously differentiable, i.e., $f\in C^{n+1}\left(\left[0,1\right]\right)$, also $V_{n}=Span{\left\{B_{0,n}\left(x\right)\;B_{1,n}\left(x\right)\;B_{2,n}\left(x\right)........B_{n,\;n}\left(x\right)\right\}}^{T}.$ If $C^{T}B$ be the best approximation fout of $V_{n},$ then $∥f-C^{T}B{∥}_{L^{2}\left[0,1\right]}\leq \frac{\alpha }{\left(n+1\right)!\sqrt{2n+3}}$, where

$$\alpha =\left|f^{n+1}\left(x\right)\right|,\;C={\left[c_{0},c_{1},c_{2},...............,c_{n}\right]}^{T}.$$

Let the exact and the approximate solutions be u(x) and ${\tilde{u}}_{n}\left(x\right)$, respectively,

(19) $${\tilde{u}}_{n}\left(x\right)=\sum_{j=1}^{n-1}c_{j\;}\varphi_{j}\left(x\right)$$

provided the functions $\varphi_{j}\left(x\right)$ are linearly independent. For detailed demonstration on convergence, we refer our previous work [10,11]

(20) $$\left|u\left(x\right)-{\tilde{u}}_{n}\left(x\right)\right|\to 0\;\text{as}\;n\to \infty ,\;0<x<1.$$

The sequence of estimated eigen-pairs converge to the precise integrals in (20) as the number of degrees of freedom grows indefinitely. This compels an uniform rate of convergence at every point in the domain. The eigen solution Eq. (19) must be used to optimize (minimize) the residual error

(21) $$e\left(x\right)=u-\tilde{u}.$$

Now we move on to demonstrate a theorem for the convergence of the suggested approach.

Let the series solution of (4) converges to the exact ones. Let ${\tilde{u}}_{n}\left(x\right)$ and ${\tilde{u}}_{m}$ be the exact and estimated solutions of Eq. (4)

(22) $$u\left(x\right)\;=\sum_{j=0}^{\infty }c_{j}\varphi_{j}\left(x\right),$$

$${\tilde{u}}_{n}\left(x\right)=\;\sum_{j=0}^{n}c_{j}\varphi_{j}\left(x\right),$$

and

$${\tilde{u}}_{m}=\sum_{j=0}^{m}c_{j}\varphi_{j}\;\text{with}\;n\geq m.$$

Then

$${\langle u\left(x\right)\;,\;\;u_{n}\left(x\right)\rangle }_{\theta (x)}={\langle u\left(x\right)\;,\;\;\sum_{j=0}^{n}c_{j}\varphi_{j}(x)\;\rangle }_{\theta (x)}=\sum_{j=0}^{n}{\bar{c}}_{j}{\langle u\left(x\right)\;,\;\;\varphi_{j}(x)\;\rangle }_{\theta (x)}=\sum_{j=0}^{n}{\bar{c}}_{j}c_{j}=\sum_{j=0}^{n}{\left|c_{j}\right|}^{2}$$

which proves that $u_{n}\left(x\right)$ is a Cauchy sequence in the complete Hilbert space[0,1]and hence converges. Thus it yields

$$\;∥u_{n}\left(x\right)-u_{m}\left(x\right){∥}_{\theta \left(x\right)}^{2}=\sum_{j=m+1}^{n}{\left|c_{j}\right|}^{2}$$

and so utilizing Bessel's inequality [39], stated in Eq. (8), $\sum_{j=\;0}^{\infty \sum }{|c_{j}|}^{2}$ is convergent which produces,as. We assume $\;\tilde{u}{\;}_{n}\left(x\right)\;$converges to some limits say ψ(x)

(23) $$\langle \psi \left(x\right)-u\left(x\right),\;\varphi_{j}{\left(x\right)}_{\theta \left(x\right)}\rangle ={\langle \psi \left(x\right),\varphi_{j}\left(x\right)\rangle }_{\theta \left(x\right)}-{\langle u\left(x\right),\varphi_{j}\left(x\right)\rangle }_{\theta \left(x\right)}=Lt{\;}_{n\to \infty }{\langle u_{n}\left(x\right),\varphi_{j}\left(x\right)\rangle }_{\theta \left(x\right)}-c_{j}=0.$$

The above result in (23) proves that$\;\sum_{j=0}^{\infty }c_{j}\varphi_{j}\left(x\right)$ converges to u(x) [40].

## Numerical illustrations

To assess the competency of the suggested method, four linear eigenvalue problems with two classes of boundary conditions have been worked out. The eigenvalues computed by the current technique are compared with the various numerical and analytical techniques [1,2,14,26] available in the literature. The Galerkin method is tested with finite intervals from eight to twelfth orders along with regular endpoint boundary conditions.

Example 1 We study the eighth order Sturm-Liouville problem [26]

(24) $$\frac{d^{8}u}{dx^{8}}=\lambda^{2}u$$

(24a) $$u\left(0\right)=u^{\prime \prime }\left(0\right)=u^{\left(4\right)}\left(0\right)=u^{\left(6\right)}\left(0\right)=0,$$

(24b) $$u\left(1\right)=u^{\prime \prime }\left(1\right)=u^{\left(4\right)}\left(1\right)=u^{\left(6\right)}\left(1\right)=0,$$

(25) $$\text{with}\;\text{exact}\;\text{eigenvalues}\;\lambda_{k}^{2}={\left(k\pi \right)}^{8};\;k=1,2,3,\ldots \ldots \ldots$$

The relative errors of the first five eigenvalues using 15 Bernstein polynomials (*n*=15) converge and match with the Magnus method [26] in the interval shown in Table 1. If the number of polynomials is increased even further, stable convergence is maintained. Our current method can accurately determine the first eigenvalues of the problem under investigation.

**Table 1:** Eigenvalues and relative errors for Example 1

Eigen index *k*	$\lambda_{Exact}$	Gal. MWR Bernstein polyn. $\lambda_{Gal}$	Relative error $|\frac{\lambda_{Gal}-\lambda_{Exact}}{\lambda_{Exact}}|$	Relative error [26]
1	9488.53101607057	9488.531016070571	3.83408e-16	1.91704e-15
2	2429063.94011407	2429063.9401140669	1.341928e-15	5.21435e-14
3	62254251.996439	62254251.996439036	5.98399e-16	3.43362e -13
4	621840368.669201	621840368.66999370	1.27483e-12	2.896264e-12
5	3706457428.15257	3706457428.4003993	6.68642e-11	4.11626e -11

Example 2 We investigate the linear stability of the steady state response in an electrohydrodynamic convection model in a layer situated toward the normal mode of perturbations [1] as defined by [3] and placed between the walls at x=±0.5.

(26a) $${\left(D^{2}-a^{2}\right)}^{4}\;u-La^{4}u+Ra^{2}\left(D^{2}-a^{2}\right)u=0,\;x\in \left(-0.5,0.5\right).$$

The boundary conditions for derivatives of even order are illustrated as

(26b) $$u=D^{2}u=D^{4}u=D^{6}u=0\;,\;at\;\;\;x=\pm 0.5.$$

The general solution can be found using the direct method by determining the determinant of the characteristic equation and being subjected to the multiplicity of the roots of the equation relevant to the problem [3]. Here *R* refers to the Rayleigh number, a and Lsatisfy the “ellipticity” condition given by $a^{4}-L\geq 0$ [3].

The authors [3] applied direct analytical/numerical schemes and thus completed the investigation of this problem. It is evident from their effort that the Rayleigh number occurs as the lowest one which is real and positive i.e.,R>0,and satisfies the eigenvalue problem (26a). If *a, L*, R≠0, the discussions of a multiplicity of the eigenvalues become difficult. So, the use of the direct analytical method is quite incomprehensible, and an alternative numerical method is sought.

Following the above facts, the authors’ in [3] applies analytical method and Chebychev and shifted Legendre polynomials spectral methods to formulate the Eq. (26a) explicitly. The authors’ used $D^{2}$ strategy worked out in their previous article [12].

**Fig. 1:**
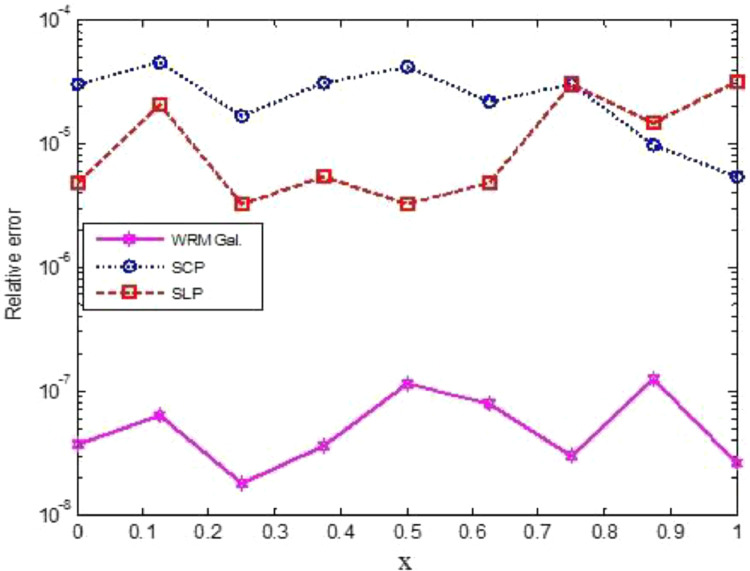
Relative errors using Galerkin MWR, Chebyshev spectral Galerkin and Legendre spectral Galerkin for Example 2.

The numerical evaluations of the first nine critical Rayleigh numbers for various spectral methods (SCP, SLP, CC) as well as the present method and two sets of L and a are exhibited in Table 2. Note that SCP, SLP, and CC imply respectively the shifted Chebyshev polynomial method, the shifted Legendre polynomial method and Chebyshev collocation method [3]. Relative errors have been shown and compared with various numerical approaches in Table 2. We noticed that compared to the Chebyshev and Legendre Spectral (SCP and SLP) approaches, the relative errors computed by our current method are significantly smaller in magnitude. All the nine critical Rayleigh numbers computed by our proposed scheme converge on the analytical ones with fewer degrees of polynomials (*n*=10). However, the parameter *R* achieved by the collocation method is lowered severely in the CC method [3]. From the comparison, we conclude that our present scheme is much more efficient and competes well with the other available techniques. Graphical representations of relative errors are shown in Fig. 1. Moreover, it is eminent that non-normality is accountable for a high spectral sensitivity. The measures of a non-normality of a complex square matrix signify as non-normality ratio H(A) and can be measured by the formula given as [3]:

(27) $$H\left(A\right)≔{\left(\rho \left(A^{*}A-AA^{*}\right)\right)}^{\frac{1}{2}},$$

where $A^{*}$ stands for conjugate transpose of A andρ(A) stands for the Frobenius norm of A. An important evaluation for H(A)

$$0\leq H\left(A\right)\leq 2{\;}^{\frac{1}{4}},\;\text{with}\;H\left(A\right)=0\;\text{if}\;A\;\text{is}\;\text{normal},\;i.e.,\;A^{*}A-AA*=0.$$

**Table 2:** Lists of calculated Rayleigh numbers and relative errors for different values of a and Lapplying various numerical techniques for Example 2.

*a*	*L*	[3]	Bernstein *Gal.* *n=10*	$R_{SCP}$ *N*=6	$R_{SLP}$ *N*=9	CC$2^{4}\leq N\leq 2^{8}$	Rel error Gal. MWR Bernstein	Rel error $R_{SCP}$	Rel error $R_{\text{SLP}}$
2	0	667.0098	667.0098	667.030	667.013	667.0092	3.648 ×$10^{-8}$	3.0284 ×$10^{-5}$	4.7975 ×$10^{-6}$
$\sqrt{4.92}$	0	657.5133	657.5133	657.543	657.527	657.5133	6.345 ×$10^{-8}$	4.5170 ×$10^{-5}$	2.0836 ×$10^{-5}$
3	0	746.5276	746.5276	746.54	746.530	746.5276	1.802 ×$10^{-8}$	1.6610 ×$10^{-5}$	3.2149 ×$10^{-6}$
2	1	666.7214	666.7214	666.742	666.725	666.7214	3.584 ×$10^{-8}$	3.0897 ×$10^{-5}$	5.3996 ×$10^{-6}$
$\sqrt{4.92}$	1	657.1806	657.1807	657.208	657.192	657.1892	1.151×$10^{-7}$	4.1693 ×$10^{-5}$	1.7347 ×$10^{-5}$
3	1	746.0506	746.0506	746.067	746.053	746.0506	7.495 ×$10^{-8}$	2.1982 ×$10^{-5}$	3.2169 ×$10^{-6}$
2	10	664.1258	664.1258	664.146	664.129	664.1258	3.003 ×$10^{-8}$	3.0416 ×$10^{-5}$	4.8184 ×$10^{-6}$
$\sqrt{4.92}$	10	654.1866	654.1866	654.193	654.177	654.1866	1.234 ×$10^{-7}$	9.7831 ×$10^{-6}$	1.4675 ×$10^{-5}$
2	16	662.3954	662.3954	662.399	662.416	35.8873	2.613 ×$10^{-8}$	5.4348 ×$10^{-6}$	3.1099 ×$10^{-5}$

**Fig. 2:**
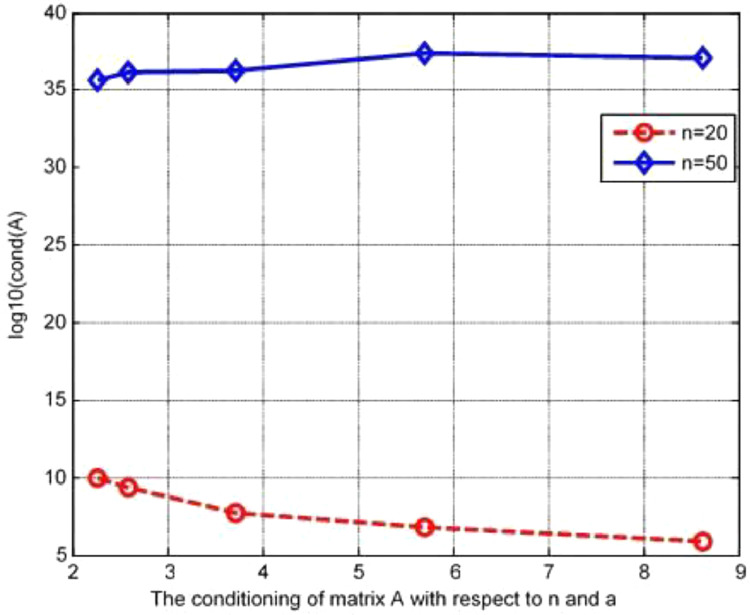
The conditioning of matrix A with respect to the wave number.

In this example, the non-normality ratio for the eighth order derivative matrix attained by our proposed Galerkin MWR executing the formula given in Eq. (27) is around 0.0009. Moreover, this ratio is around 0.9 with respect to the eigenvalue and does not depend upon the degree of polynomials in the range 10<n<40. Fig. 2 displays the curve *log* (Cond(*A*)) versus 2 <a<9 for the degree of polynomials *n*= 20 and *n*=50 of Example 2. It has been observed that for *n*=50, the condition numbers of the eigenvalues for different values of wave numbers using twenty polynomials vary between $O(10^{5})\;$to$\;O(10^{8})$ and using fifty polynomials vary between $O(10^{35})$ to $O(10^{37})$ and are growing for the increasing a. For *n*=20, the first curve moderately decreases as the wave numbers increase. It is noticed that forn=50,thesecond curve is almost horizontal for 6≤a≤9. This shows that for larger *n* and for various distinct values of *a*, the condition number$\left(|\frac{max.\;eigenvalue}{min.\;eigenvalue}|\right)$ of the Rayleigh numbers does not worsen, indicating that the existing technique is stable.

Fig. 1 demonstrates that the suggested approach's errors for the nine critical Rayleigh numbers are of order $10^{-8}$ and, (n=10), whereas the smallest errors reported by [3] by exploiting those of Chebyshev and Legendre Spectral (SCP and SLP) methods is of order $10^{-6}$. As a result, the current technique achieves precisely accurate lower Rayleigh numbers.

Example 3 We consider the eighth order ordinary differential equation worked out in [1]

(28a) $$\left(D^{8}-j_{1}D^{6}+j_{2}D^{4}-j_{3}D^{2}+j_{4}\right)w\left(x\right)+i\mu \left(-j_{5}D^{6}+j_{6}D^{4}-j_{7}D^{2}+j_{8}\right)w\left(x\right)+RA^{2}\left(D^{2}-A^{2}\right)w\left(x\right)-i\mu RA^{2}w\left(x\right)=0,\;0<x<1.$$

It is necessary to enforce the corresponding free-free boundary conditions given by

(28b) $$w^{\left(2i\right)}\left(0\right)=w^{\left(2i\right)}\left(1\right)=0,\;i=0,1,2,3,$$

where

$$j_{1}=4A^{2},\;\;j_{2}=6A^{4}-\mu^{2}\left(2p_{1}+1\right)+T,\;\;j_{3}=4A^{6}-2A^{2}\mu^{2}\left(2p_{1}+1\right)+A^{2}T,$$

$$j_{4}=A^{8}-A^{4}\mu^{2}\left(2p_{1}+1\right),\;j_{5}=\left(p_{1}+2\right),j_{6}=3A^{2}\left(p_{1}+2\right),\;j_{7}=3A^{4}\left(p_{1}+2\right)\mu^{2}p_{1}+p_{1}T,$$

$$j_{8}=A^{6}\left(2p_{1}+2\right)-A^{2}\mu^{2}p_{1}.$$

We consider the eighth-order differential eigenvalue problem (1) in which σ=iμ is purely imaginary and takes the form as given in (28a). Numerical results are obtained using *n=*10, $p_{1}=0.025\;$for the first six critical values of problem 3 are displayed in Table 3.

**Table 3:** Estimated values of critical Rayleigh numbers utilizing for Example 3.

N	T	$A_{Gal}$ current	$A_{FDM}$[1]	$A_{C}$[2]	$\mu_{GAL}$ current	$\mu_{FDM}$[1]	$\;\mu_{C}$[2]	$R_{Gal}$ current	$\;R_{FDMMM}$[1]	$\;\;R_{C}$[2]
8	1.681 $\times \;10^{4}$	2.270	2.270	2.270	101.389	101.383	101.4	1388	1387.2	1388
8	1.68$1\;\times \;10^{5}$	2.594	2.594	2.594	307.901	307.881	307.9	1693.9	1693.9	1694
8	1.681 $\times \;10^{6}$	3.710	3.710	3.710	816.745	816.820	816.8	3436	3436.1	3436
8	1.681 $\times \;10^{7}$	5.698	5.698	5.698	1930.002	1930.237	1930.0	11023.1	11023.1	11020
8	1.681 $\times \;10^{8}$	8.626	8.626	8.626	4325.888	4326.506	4330.0	43679.9	43673.1	43680

**Fig. 3:**
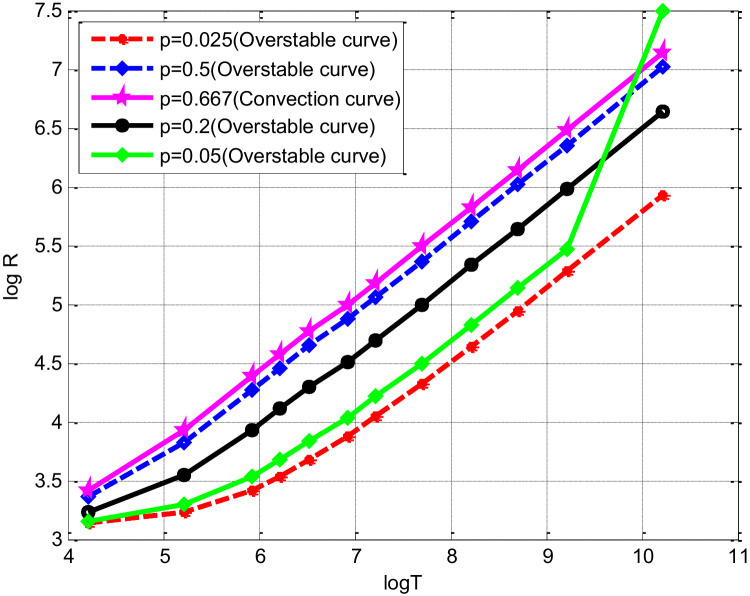
$\left(R_{C},\;T\right)$- relation for different Prandtl number p for convection and over stability prediction.

Following [1,2], the minimum values of $\;R_{Gal}$ and the associated values of $A_{Gal}$ and $\sigma (=i\mu_{Gal})$ are computed for five distinct values of the Taylor number T. For given values of T, A and μ, each eigenvalue obtained by our present method $R_{Gal}$ is real whereas the same are complex as demonstrated in [1,2]. The results are depicted in Table 3 in comparison with other numerical techniques show that the present method is compatible and much more efficient. The minimum eigenvalue $R_{Gal}$ is produced corresponding to the wave number $A_{Gal}=2.270$ and σ=101.389. The values of $R_{Gal},$ $A_{Gal}$ and $\sigma (=i\mu_{GAL})$ are compared to the corresponding values of $R_{C},$ $A_{C}$ and $\sigma_{C}\left(=i\mu_{C}\right)$ and $R_{FDM},\;A_{FDM,\;}\sigma_{FDM}\;$which are demonstrated in [2] and [1], respectively. It is observed that in accordance with the result given in [2] along the various over stable solutions $R\to constant\;T^{\frac{1}{2}}$ as T→∞. The explicit form of the asymptotic behaviors of the Rayleigh number and the wave number of the associated disturbance for the onset of over stability may be noted; they are illustrated in detail in [2]. Note that asymptotic relations are valid only for $p^{2}T\to \infty ,\;$not for T→∞, a distinction which is important when p→0. In Fig. 3, we illustrate the $(R_{Gal},\;T)-$ relations for a spinning bottom layer of fluid heated below. It is noticed that for each value of p<0.667, the instability sets in as ordinary cellular convection for T less than a certain $T^{\left(p\right)}$ while it sets in as over stability for $T>T^{p}$.The values of $T^{\left(p\right)}$ can be determined using the conditions given for critical and minimum Rayleigh numbers discussed in [2].

Example 4 We examine the Sturm-Liouville problem of tenth order [14]. To which the various curves are shown

(29a) $$\frac{d^{10}u}{dx^{10}}=-\lambda u\left(x\right).$$

The boundary conditions are:

(29b) $$\left\{ \begin{array}{c} u\left(0\right)=u^{2}\left(0\right)=u^{4}\left(0\right)=u^{6}\left(0\right)=u^{8}\left(0\right)=0\\ \;u\left(\left(\pi \right)\right)=u^{2}\left(\pi \right)=u^{4}\left(\pi \right)=u^{6}\left(\pi \right)=u^{8}\left(\pi \right)=0 \end{array}\right.\;\;\;\;.$$

The first six eigenvalues utilizing Galerkin MWR exploiting for Eqs. (29a) and (29b) using Bernstein polynomials along with their absolute error with ADM [14] are displayed in Table 4. Table 4 reveals that the absolute errors |$\lambda_{ADM}-\lambda_{Gal}$ | for all six eigenvalues are exceedingly small i.e., 1.0e+00. This shows that our proposed method agrees well with ADM [14].

**Table 4:** The absolute errors for first six eigenvalues of Example 4.

*k*	Gal. MWR Bernstein polyn. $\lambda_{k}$	ADM [14]$\lambda_{k}$	Absolute error $|\lambda_{ADM}-\lambda_{GAL}|$
1	1.000000000000000	1.0000000000000000	$1.0\;\times \;10^{-0}$
2	1024.000000000000000	1024.0000000000000000	$1.0\;\times \;10^{-0}$
3	59049.0000000000000000	59049.0000000000000000	1.0$\times 10^{-0}$
4	1048576.000000000000000	1048576.000000000000000	$\;1.0\;\times \;10^{-0}$
5	9765625.000000000000000	9765625.000000000000000	$\;1.0\;\times \;10^{-0}$
6	604666176.000000000000	604666175.99999945213	1.049 $\times 10^{-0}$

Example 5 The tenth order eigenvalue problem studied in [1] is considered

(30a) $$\left(D^{10}-k_{1}D^{8}+k_{2}D^{6}-k_{3}D^{4}+k_{4}D^{2}-A^{10}\right)w\left(x\right)+RA^{2}\left(D^{4}+k_{5}D^{2}+A^{4}\right)w\left(x\right)=0,\;0<x<1,$$

$$k_{1}=5A^{2}+2Q,\;k_{2}=10A^{4}\;+6A^{2}Q+T+Q^{2},\;\;k_{3}=10A^{6}+6A^{4}Q+2A^{2}T+A^{2}Q^{2},$$

$$k_{4}=5A^{8}+2A^{6}Q+A^{4}T,\;k_{5}=2A^{2}+Q.$$

The corresponding free-free boundary conditions are given as follows:

(30b) $$w^{\left(2i\right)}\left(0\right)=w^{\left(2i\right)}\left(1\right)=0,\;i=0,1,2,3,4.$$

According to [2], here we note that the non-dimensional number Q plays for problem involving magnetic fields the same role which the Taylor numberT plays for problems involving rotation. From Eq. (2) it follows that, instability appears first for the lowest mode n=1and the equivalent formula for R is [2]

(31) $$R=\pi^{4}\frac{\left(1+x\right)\left\{{\left[{\left(1+x\right)}^{2}+Q_{1}\right]}^{2}+T_{1}\left(1+x\right)\right\}}{x\left[{\left(1+x\right)}^{2}+Q_{1}\right]}$$

where the relationship between Q, $Q_{1}\;$and T, $T_{1}$ are

(31a) $$\frac{A^{2}}{\pi^{2}},\;Q_{1}=\frac{Q}{\pi^{2}}\;\text{and}\;T_{1}=\frac{T}{\pi^{4}}.$$

**Fig. 4:**
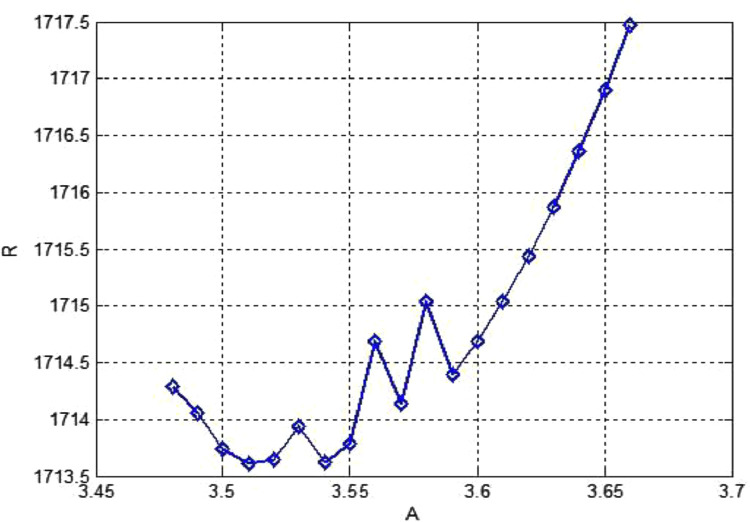
The dependence of Rayleigh number$\;R_{Gal}$ for the onset of instability on the wave number for some preassigned values of Taylor number (T) and magnetic field intensity(Q).

**Fig. 5:**
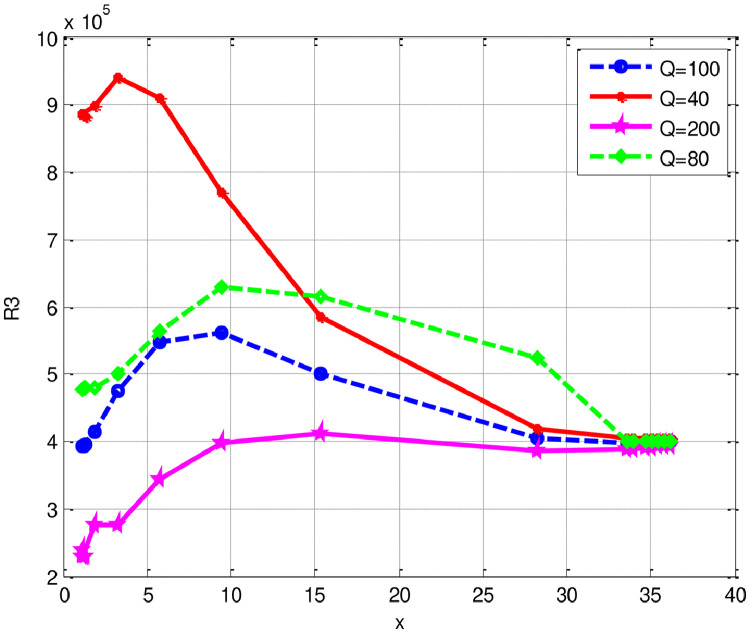
Estimated values of Critical Rayleigh numbers for Example 5.

R can be evaluated directly as a function of x(in accordance with Eq. (31) and locate the minimum numerically [2]. The eigenvalue problem in Eqs. (30a) and (30b) is solved using the formulation illustrated in this section. To compare the computed results demonstrated in [2]. Computed results of $R_{Gal\;}$for the corresponding values of the wave number $A_{Gal}$ obtained for $T_{1}=\;1000$ and $T_{1}$=10,000 listed in Tables 5(a) and 5(b). Moreover, numerical values for $T_{1}=10$, $Q_{1}=1\;$are depicted in Fig. 4. The solution of the problem (2) presents some unpredicted features depicted in Figs. 4 and 5.

**Table 5a:** Estimated values of critical Rayleigh numbers for $T_{1}$=1000 for Example 5.

*n*	$Q_{1}$	Present method Bernstein polyn.		FDM [1]		Analytical [2]	
		$\;R_{Gal}$	$A_{Gal}$	$R_{1}$	$A_{1}$	$\;R_{C}$	$\;A_{C}$
10	10	2.016 $\times \;10^{4}$	7.90	2.016 $\times \;10^{4}$	7.90	2.016 $\times \;10^{4}$	7.90
10	50	1.605 $\times \;10^{4}$	4.50	1.604 $\times \;10^{4}$	4.50	1.605 $\times \;10^{4}$	4.50
10	100	1.-952 $\times \;10^{4}$	5.23	1.951 $\times \;10^{4}$	5.22	1.952 $\times \;10^{4}$	5.23
10	500	6.378 $\times \;10^{4}$	7.47	6.377 $\times \;10^{4}$	7.46	6.380 $\times \;10^{4}$	7.47
10	1000	1.192 $\times \;10^{5}$	8.52	1.192 $\times \;10^{5}$	8.52	1.192 $\times \;10^{5}$	8.52
10	10000	1.065 $\times \;10^{6}$	12.80	1.065 $\times \;10^{6}$	12.80	1.065 $\times \;10^{6}$	12.80
10	50000	5. 129 $\times \;10^{6}$	16.82	5.128 $\times \;10^{6}$	16.82	5.129 $\times \;10^{6}$	16.82
10	100000	1.005 $\times \;10^{7}$	18.94	1.015 $\times \;10^{7}$	18.94	1.015 $\times \;10^{7}$	18.94

**Table 5b:** Estimated values of Critical Rayleigh numbers for $T_{1}$=10,000 for Example 5.

Eigen index	Degree of polyn.	$Q_{1}$	Present method		FDM [1]		Analytical [2]	
			$\;R_{Gal}$	$A_{Gal}$	$R_{1}$	$A_{1}$	$\;R_{C}$	$A_{C}$
1	8	10	8.979 $\times \;10^{4}$	12.59	$8.977\;\times \;10^{4}$	12.59	$8.979\;\times \;10^{4}$	12.59
2	8	50	8.550 $\times \;10^{4}$	11.40	8.548 $\times \;10^{4}$	11.40	8.550 $\times \;10^{4}$	11.40
3	8	100	8.118 $\times \;10^{4}$	3.68	8.116 $\times \;10^{4}$	3.68	8.118 $\times \;10^{4}$	3.68
4	8	500	5.544 $\times \;10^{4}$	3.91	$\;5.542\;\times \;10^{4}$	3.91	5.544 $\times \;10^{4}$	3.91
5	8	1000	7.745 $\times \;10^{4}$	6.50	$\;7.543\;\times \;10^{4}$	6.51	$\;7.545\;\times \;10^{4}$	6.50
6	8	10000	1.267 $\times \;10^{5}$	7.98	$\;1.267\;\times \;10^{5}$	7.98	1.26$1\;\times \;10^{5}$	7.98
7	8	50000	1.067 $\times \;10^{6}$	12.80	1.066 $\times \;10^{6}$	12.77	1.067 $\times \;10^{6}$	12.80
8	8	100000	1.011 $\times \;10^{7}$	18.94	1.015 $\times \;10^{7}$	18.93	1.015 $\times \;10^{7}$	18.94

We have noticed from the results in Table 5(b) that the actual minima depend on $Q_{1}$ and $T_{1}$ .Two minima at $A_{Gal}=3.51$, minimum value of $R{\;}_{Gal}=1713.60877$ and at $A_{Gal}=3.54$, minimum value of $R_{Gal}=1713.6188$.

For $Q_{1}=100$, $T_{1}=100000$,$\;R_{Gal}=397672.43782,\;A_{Gal}=18.2,$ $R_{Gal}=$393296.2090005, $A_{Gal}=3.37$.

It has been observed that for $Q_{1}=80$, $T_{1}=100000$, usingn=10, the least values of $R_{Gal}$ and the associated values of $A_{Gal}$ [1] are shown below:

$$R_{Gal}=399797.1868793\;\text{with}\;A_{Gal}=18.3,\;\;R_{Gal}=478101.133947\;\text{with}\;A_{Gal}=3.38.$$

The dependence of the Rayleigh number $R_{Gal}$ for the onset of instability on the wave number when $T_{1}=10^{5}$ and $Q_{1}=40,\;80$, 100 and 200. The abscissa is related to the wave number as given in (31a). We observe the occurrence of two minima and their relative disposition displayed in Fig. 5. In other words, if we start with an initial situation in which $T_{1}=10^{5}$ and no magnetic field exists; gradually strengthen the magnetic field.

Example 6 We studied the following twelfth order eigenvalue problem [1]

(32a) $$\begin{array}{ccc} & & \left(D^{12}-l_{1}D^{10}+l_{2}D^{8}-l_{3}D^{6}+l_{4}D^{4}-l_{5}D^{2}+l_{6}\right)w\left(x\right)+i\left(-l_{7}D^{10}+l_{8}D^{8}-l_{9}D^{6}+l_{10}D^{4}-l_{11}D^{2}+l_{12}\right)w\left(x\right)\\ & & \;+iRA^{2}\left(-l_{16}D^{4}+l_{17}D^{2}-l_{18}\right)w\left(x\right)=0. \end{array}$$

The corresponding free-free boundary conditions illustrated in [1]:

(32b) $$\;w^{\left(2i\right)}\left(0\right)=w^{\left(2i\right)}\left(1\right)=0;\;i=0,1,2,3,4,5.$$

The coefficients $l_{i}\left(i=1,2,3,........,18\right)$ are given by

$$l_{1}=6A^{2}+2Q,$$

$$l_{2}=15A^{4}+8QA^{2}+Q^{2}-\left[2p_{2}+2p_{1}\left(1+p_{2}\right)+\left(1+p_{2}\right)+{\left(1+p_{2}\right)}^{2}\right]\mu^{2}+T$$

$$l_{3}=20A^{6}+120QA^{4}-4\left[2p_{2}+2p_{1}\left(1+p_{2}\right)+{\left(1+p_{2}\right)}^{2}\right]\mu^{2}A^{2}+3TA^{2}+2Q^{2}A^{2}-2\left(p_{1}+p_{2}+p_{1}p_{2}\right)\mu^{2},$$

$$\begin{array}{ccc} l_{4} & = & 15A^{8}+8QA^{6}-6\left[2p_{2}+2p_{1}\left(1+p_{2}\right)+{\left(1+p_{2}\right)}^{2}\right]\mu^{2}A^{2}+3TA^{2}+2Q^{2}A^{2}-4\left(p_{1}+p_{2}+p_{1}p_{2}\right)\mu^{2}A^{2}\\ & & +\left[p_{2}^{2}+2p_{1}p_{2}\left(1+p_{2}\right)\right]\mu^{4}-p_{2}\left(2p_{1}+p_{2}\right)T, \end{array}$$

$$l_{5}=6A^{10}+2QA^{8}-4\left[2p_{2}+2p_{1}\left(1+p_{2}\right)+{\left(1+p_{2}\right)}^{2}\right]\mu^{2}A^{4}+2\left[p_{2}^{2}+2p_{1}p_{2}\left(1+p_{2}\right)\right]\mu^{4}A^{2}-p_{2}\left(2p_{1}+p_{2}\right)T\mu^{2}A^{2},$$

$$l_{6}=A^{12}-\left[2p_{2}+2p_{1}\left(1+p_{2}\right)+{\left(1+p_{2}\right)}^{2}\right]\mu^{2}A^{8}+\left[p_{2}^{2}+2p_{1}p_{2}\left(1+p_{2}\right)\right]\mu^{4}A^{4}-p_{2}\left(2p_{1}+p_{2}\right)T\mu^{2}A^{2},$$

$$l_{7}=\left(2+p_{1}+2p_{2}\right)\mu ,$$

$$l_{8}=2\left(1+p_{1}+p_{2}\right)\mu Q+5\left(2+p_{1}+2p_{2}\right)\mu A^{2},$$

$$l_{9}=10\left(2+p_{1}+2p_{2}\right)\mu A^{4}+6\left(1+p_{1}+p_{2}\right)\mu QA^{2}-\left[2p_{1}p_{2}+2p_{2}\left(1+p_{2}\right)+p_{1}{\left(1+p_{2}\right)}^{2}\right]\mu^{3}+3\left(p_{1}+2p_{2}\right)T\mu +p_{1}\mu Q^{2},$$

$$l_{10}=10\left(2+p_{1}+2p_{2}\right)\mu A^{6}+6\left(1+p_{1}+p_{2}\right)\mu QA^{4}+2\left(p_{1}+2p_{2}\right)T\mu A^{2}-3\left[2p_{1}p_{2}+2p_{2}\left(1+p_{2}\right)+p_{1}{\left(1+p_{2}\right)}^{2}\right]\mu^{3}A^{2},$$

$$\begin{array}{ccc} l_{11} & = & 5\left(2+p_{1}+2p_{2}\right)\mu A^{8}+2\left(1+p_{1}+p_{2}\right)\mu QA^{6}-3\left[2p_{1}p_{2}+2p_{2}\left(1+p_{2}\right)+p_{1}{\left(1+p_{2}\right)}^{2}\right]\mu^{3}A^{4},\\ & & -3\left[2p_{1}p_{2}+2p_{2}\left(1+p_{2}\right)+p_{1}{\left(1+p_{2}\right)}^{2}\right]\mu^{3}A^{2}+\left(p_{1}+2p_{2}\right)T\mu A^{4}+p_{1}p_{2}^{2}\mu^{5}-p_{1}p_{2}^{2}\mu^{3}T, \end{array}$$

$$l_{12}=\left(2+p_{1}+2p_{2}\right)\mu A^{10}-\left[2p_{1}p_{2}+2p_{2}\left(1+p_{2}\right)+\;p_{1}{\left(1+p_{2}\right)}^{2}\right]\mu^{3}A^{6}+p_{1}p_{2}^{2}\mu^{5}A^{2},$$

$$l_{13}=3A^{2}+Q,\;l_{14}=3A^{4}+QA^{2}-p_{2}\left(2+p_{2}\right)\mu^{2},\;l_{15}=A^{6}-p_{2}\left(2+p_{2}\right)\mu^{2}A^{2},$$

$$l_{16}=\left(1+2p_{2}\right)\mu ,$$

$$l_{17}=2\left(1+2p_{2}\right)\mu A^{2}+p_{2}\mu Q,$$

$$l_{18}=\left(1+2p_{2}\right)\mu A^{4}-p_{2}^{2}\mu^{3}.$$

Here $Q_{1}=\frac{Q}{\pi^{2}}$, $\;T_{1}=\frac{T}{\pi^{4}}\;$and$\;\sigma =\frac{\sigma_{1}}{\pi^{2}}$.

The numerical results for $R_{Gal}\;$using Eqs. (32a) and (32b) are computed using Galerkin MWR for increasing Taylor numbers and the same magnetic field strength are displayed in Tables 6(a), 6(b), and 6(c). Similar outcomes are also described in [1]. Tables 6(a), 6(b), and 6(c) show that our current method produces extremely similar results, except for Table 6(c), where the results are slightly deviated from the reported results. We conclude that for higher Taylor numbers, the computed results become slightly less accurate than those of [1].

**Table 6a:** Computed values of R, $A_{1}\;$andσ for$\;T_{1}=1000\;$for Example 6.

		Present method			FDM [1]			Analytical [2]		
*n*	$Q_{1}$	*R*	A	σ	$R_{1}$	$A_{1}$	$\sigma_{1}$	$R_{c}$	$A_{c}$	$\sigma_{c}$
16	10	7053	4.56	53.52i	7053	4.55	53.61i	7053	4.56	53.52i
38	100	35398	6.63	30.13i	35390	6.62	30.13i	35402	6.63	30.12i
18	500	50156	7.20	18.44i	50140	7.20	18.43i	50156	7.20	18.44i

**Table 6b:** Computed values of R, andσ, $T_{1}=100,000\;$of Example 6.

		Present results			FDM [1]			Analytical [2]		
*n*	$Q_{1}$	R	A	σ	$R_{1}$	$A_{1}$	$\sigma_{1}$	$R_{c}$	$A_{c}$	$\sigma_{c}$

10	10	12839	6.03	140.80*i*	12839	6.02	53.61*i*	12840	6.03	140.80*i*
38	50	26772	7.01	12.43*i*	35390	7.05	120.43*i*	26790	7.05	120.4*i*
38	500	155675	9.84	61.95*i*	155635	9.88-	61.45*i*	155700	9.88	61.95*i*

**Table 6c:** Computed values of R,$\;A_{1\;},\;$andσ for$\;T_{1}=1000000\;$of Example 6.

		Present Gal. MWR			FDM [1]			Analytical [1]		
*n*	$Q_{1}$	R	*A*	σ	$R_{1}$	$A_{1}$	$\sigma_{1}$	$R_{c}$	$A_{c}$	$\sigma_{c}$
38	10	36495	8.35	342.45i	36390	8.35	341.5i	36500	8.35	342.1*i*
40	500	189238	11.50	236.14i	189209	11.55	236.14i	189300	11.54	236.3*i*
12	1000	3,26460	12.64	202.34i	326,666	12.64	202.45i	327100	12.64	202.5*i*

## Conclusions

In this study, we attempt a novel numerical method for computing approximate eigenvalues/critical numbers of regular eighth, tenth and twelfth order eigenvalue problems. All the derivative boundary conditions have been incorporated directly in the residual equation without reducing the order of the derivatives, with the help of orthogonal Bernstein polynomials as bases.

Our results by Galerkin MWR for 8th order problems are demonstrated in Tables 1 and 2. In terms of computational efficiency, the numerical results reveal that the presented approach performs well with the Magnus expansion method. The comparability of the calculated Rayleigh numbers with the analytical solutions reveals a good estimation as it guarantees compatibility with the other existing methods. From Tables 3, 4, Table 5 and Table 6, it has been noticed that, estimated critical Rayleigh numbers corresponding to the fixed wave numbers are much closer to [1,2]. Thus, the existing method shows a good performance and achieves high precision when applied to numerous problems presented in this study. Although the estimated critical values of *A* differ slightly from those in Ref. [1], Tables for twelfth order eigenvalue problems reveal that results produced are lower, implying that over stability is the cause of instability. This method produces a sparse co-efficient matrix with a symmetric banded matrix, which reduces computation cost and results in a well-conditioned matrix. Hence implementing the boundary conditions is much simpler and easier in these problems. The linear stability of the electro-hydro dynamic (EHD) problem was efficiently solved by exploiting Galerkin MWR. Our numerical calculations confirms that Galerkin matrix behaves better than both the Chebyshev Tau and collocation matrices. The current scheme produces a more normal discretization matrix as it produces reduced non-normality ratio. Also the resulting matrix in the latter case is also symmetric and agrees well with the other published results. The stability or instability of hydrodynamic and hydro-magnetic systems can be comprehended by a set of non-dimensional parameters. A comparisons of critical Rayleigh numbers calculated by the proposed method and those of other existing techniques are made. The obtained results confirm that the proposed method possesses much more accurate results, is more expedient and competent for higher order eigenvalue problems. Despite being more precise and effective with constant coefficients than those of the problems with variable coefficients regardless of order, the current method has a drawback in that it is less accurate and efficient with variable coefficients.

## CRediT authorship contribution statement

**Humaira Farzana:** Methodology, Investigation, Software, Writing – original draft. **Samir Kumar Bhowmik:** Methodology, Formal analysis, Writing – original draft. **Md. Shafiqul Islam:** Conceptualization, Writing – review & editing.

## Declaration of Competing Interest

The authors declare that they have no known competing financial interests or personal relationships that could have appeared to influence the work reported in this paper.

## Figures and Tables

**Table:** 

ν	:	kinematic viscosity,
κ	:	thermometric conductivity,
μ	:	magnetic permeability,
σ	:	time constant (relative to dimensionless time and space coordinates),
η	:	$\frac{1}{\left(4\pi \sigma \mu \right)}$
H	:	uniform magnetic field,
d	:	depth of layer of fluid,
ρ	:	density of fluid,
Ω	:	angular velocity,
α	:	coefficient of volumetric expansion
β	:	adverse temperature gradient,
A	:	wave number,
g	:	acceleration due to gravity,
Q	=	$\frac{\mu H^{2}d^{2}}{\left(4\pi \rho \nu \eta \right)}$
T	=	$\frac{4\Omega^{2}d^{4}}{\nu^{2}}:$ the Taylor number,
R	=	$\frac{g\alpha \beta d^{4}}{\left(\kappa \nu \right)}:$ Rayleigh number,
$R_{c}$	:	Critical Rayleigh number,
$p_{1}$	:	$\frac{\nu }{\kappa }:$ Prandtl number,
$p_{2}$	=	$\frac{\eta }{\kappa },$
x	:	dimensionless vertical co-ordinate,
w	=	w(x): vertical co-ordinate.

## Data Availability

No data was used for the research described in the article.
